# Characterizing intra-tumor regions on quantitative ultrasound parametric images to predict breast cancer response to chemotherapy at pre-treatment

**DOI:** 10.1038/s41598-021-94004-y

**Published:** 2021-07-21

**Authors:** Hamidreza Taleghamar, Hadi Moghadas-Dastjerdi, Gregory J. Czarnota, Ali Sadeghi-Naini

**Affiliations:** 1 Department of Electrical Engineering and Computer Science, Lassonde School of Engineering, York University, Toronto, ON Canada; 2 Department of Medical Biophysics, University of Toronto, Toronto, ON Canada; 3 Physical Sciences Platform, Sunnybrook Research Institute, Sunnybrook Health Sciences Centre, Toronto, ON Canada; 4 Department of Radiation Oncology, Odette Cancer Centre, Sunnybrook Health Sciences Centre, Toronto, ON Canada

**Keywords:** Breast cancer, Cancer imaging, Tumour biomarkers, Tumour heterogeneity, Predictive markers, Prognostic markers, Biophysics, Cancer, Biomarkers, Oncology, Biomedical engineering

## Abstract

The efficacy of quantitative ultrasound (QUS) multi-parametric imaging in conjunction with unsupervised classification algorithms was investigated for the first time in characterizing intra-tumor regions to predict breast tumor response to chemotherapy before the start of treatment. QUS multi-parametric images of breast tumors were generated using the ultrasound radiofrequency data acquired from 181 patients diagnosed with locally advanced breast cancer and planned for neo-adjuvant chemotherapy followed by surgery. A hidden Markov random field (HMRF) expectation maximization (EM) algorithm was applied to identify distinct intra-tumor regions on QUS multi-parametric images. Several features were extracted from the segmented intra-tumor regions and tumor margin on different parametric images. A multi-step feature selection procedure was applied to construct a QUS biomarker consisting of four features for response prediction. Evaluation results on an independent test set indicated that the developed biomarker coupled with a decision tree model with adaptive boosting (AdaBoost) as the classifier could predict the treatment response of patient at pre-treatment with an accuracy of 85.4% and an area under the receiver operating characteristic (ROC) curve (AUC) of 0.89. In comparison, the biomarkers consisted of the features derived from the entire tumor core (without consideration of the intra-tumor regions), and the entire tumor core and the tumor margin could predict the treatment response of patients with an accuracy of 74.5% and 76.4%, and an AUC of 0.79 and 0.76, respectively. Standard clinical features could predict the therapy response with an accuracy of 69.1% and an AUC of 0.6. Long-term survival analyses indicated that the patients predicted by the developed model as responders had a significantly better survival compared to the non-responders. Similar findings were observed for the two response cohorts identified at post-treatment based on standard clinical and pathological criteria. The results obtained in this study demonstrated the potential of QUS multi-parametric imaging integrated with unsupervised learning methods in identifying distinct intra-tumor regions in breast cancer to characterize its responsiveness to chemotherapy prior to the start of treatment.

## Introduction

Breast Cancer is the most frequent malignancy and the leading cause of cancer-related death among women^1,2^. In 2018 more than 2 million new breast cancer cases were diagnosed, and more than 0.6 million people died from it^3^. Up to 20% of breast cancer patients are diagnosed with locally advanced breast cancer (LABC) that often presents as tumors greater than 5 cm in size, possibly with regional lymph node, skin and/or chest wall involvement^4,5^. LABC patients have a high risk of relapse and metastasis, with a 5-year survival rate of around 55%^5^. The standard treatments for LABC patients include a combination of neoadjuvant chemotherapy (NAC), followed by surgery, and if required, adjuvant radiation and/or hormonal therapies^4,6^. Response to NAC has demonstrated a high correlation to the patient survival^6–8^. However up to 40% of LABC patients do not respond to NAC, and complete pathological response is limited to only 10–30% of the patients^4,5,9–12^. Current methods for evaluating response to NAC are based on changes in tumor size in routine physical examination or anatomical imaging. However, changes in tumor size may require many weeks to months of therapy to be detectable, and in some cases, it is not evident despite a pathological response to NAC^13^. Post-surgical histopathology is the standard approach to determine tumor pathological response to NAC^7,8,14,15^. However, at that point the window to adjust the NAC or switch to a salvage treatment is already closed. Prediction of LABC response to NAC before or early after the start of treatment can facilitate changing ineffective treatments to more effective ones. A personalized treatment strategy for LABC patients is expected to improve the rate of response to neoadjuvant therapies, and the overall survival and quality of life of the patients.

Genetic approaches have recently been investigated for prediction of cancer response to treatment^16^. Specifically, analysis of circulating tumor DNA has shown promise in evaluation of breast cancer response to therapy^17,18^. Whereas such methods provide crucial scientific insights, they are invasive, relatively expensive, and require time-consuming analyses for quantification of circulating tumor DNA and gene sequencing. For monitoring and evaluating breast cancer response to NAC, functional imaging techniques including positron emission tomography (PET) and magnetic resonance imaging (MRI) have been investigated and shown promise within weeks after the treatment initiation^19–21^. However, these modalities are often expensive with long scan times and need injection of contrast agents to detect functional changes in tumor in response to treatment. Adapting an imaging modality with higher availability, lower cost, and an intrinsic source of image contrast to predict tumor response would facilitate adoption of the developed methodologies in routine clinical practice.

Ultrasound is a relatively inexpensive and portable imaging modality with a high spatial resolution and short imaging time that does not require injection of exogenous contrast agents. Quantitative ultrasound (QUS) techniques examine the frequency dependence of the radiofrequency (RF) signal backscattered from the underlying tissue to extract parameters that quantify tissue physical properties, and can be used to characterize tissue micro-structure^22^. Specifically, efficacy of the QUS parameters derived from the analysis of normalized power spectrum of RF signal, including mid-band fit (MBF), spectral slope (SS), spectral 0-MHz intercept (SI), effective scatterer diameter (ESD) and effective acoustic concentration (EAC) have been demonstrated in detecting and characterizing different abnormalities including prostate and breast cancer, intraocular tumors and cardiovascular disease^23–28^.

A number of previous studies have demonstrated that changes in QUS spectral parameters after the start of treatment could be used to detect tumor cell death^29^, and monitor breast cancer responses to chemotherapy^30–32^. Also, it has been demonstrated that compared to the QUS mean-value parameters, alterations in the textural characteristics of QUS spectral parametric maps have higher correlations to histological tumor cell death in response to chemotherapy^33^, and could be used to predict LABC tumor response to NAC as early as one week after starting the treatment^34,35^. Textural measures of the QUS parametric maps quantify the spatial relationship between local acoustic properties within the tumor and their early alterations after treatment initiation could characterize changes in response-related intra-tumor heterogeneity^35^. Sannachi et al*.* showed that a combination of QUS spectral and, textural parameters and molecular features of tumor could predict the LABC tumor response with high sensitivity and specificity^36^. In a recent study, Tadayyon et al*.* have demonstrated that a combination of QUS parameters derived from the tumor core and margin could be applied to characterize the responsiveness of LABC tumors to NAC before starting the treatment^37^. In particular, their study highlighted the importance of spatial heterogeneity within tumor core and margin in characterizing tumor aggressiveness and predicting its likelihood of response to standard chemotherapy at pre-treatment.

Imaging-based characterization of distinct intra-tumor regions has been shown efficacious for characterizing malignancies and predicting their therapy outcome^38–40^. Previous studies have investigated the potential of imaging tumor habitats in characterizing the lung cancer^41^, head and neck cancer^42^, and the breast cancer^43^ and demonstrated that the characteristics of such habitats can be associated with the treatment response and patient survival. Intra-tumor regions evident on imaging can be linked to differential tumor biology and micro-structure, including clusters of heterogenous cancer cells, activated molecular pathways, calcification foci, hypoxic or necrotic/apoptotic areas, and regions with different perfusion and metabolic activities^27,35,44–46^. A study by Byra et al*.* has demonstrated that features of intra-tumor regions identified using QUS maps of homodyned K distribution parameters could be used to differentiate benign and malignant breast lesions^40^. Another study by Wu et al*.* has showed the potential of characterizing intra-tumor regions on MRI in predicting pathological response of breast tumors to chemotherapy^47^. Despite its demonstrated potential for tissue characterization in various diagnostic and prognostic applications, to our knowledge, no previous work has applied QUS parametric imaging to quantify distinct intra-tumor regions for therapy response prediction.

This study investigated the efficacy of QUS spectral multi-parametric imaging in characterizing LABC intra-tumor regions to predict tumor response to NAC before the start of treatment. QUS spectral parametric images were generated using the ultrasound data acquired from 181 LABC patients at pre-treatment. The dataset was randomly partitioned into a training set (70%) and an independent test set (30%). A hidden Markov random field (HMRF) expectation maximization (EM) algorithm was applied to identify distinct intra-tumor regions on QUS multi-parametric images^48^. Several features were extracted from the segmented regions on different parametric maps within the tumor core and margin to characterize each tumor. The features were analyzed using a multi-step feature ranking and selection process to construct an optimal QUS biomarker consisting of four features for response prediction. For comparison, the features extracted from the unsegmented tumor core and margin were also analyzed and applied for predicting the therapy response. A decision tree model with adaptive boosting (AdaBoost) was adapted for classifying patients into responders and non-responders at pre-treatment. The patient responses to NAC identified after their surgery using standard clinical and pathological criteria were used as the ground truth to evaluate the performance of prediction models. Results indicated that the model with the developed biomarker could predict the NAC response of patients of the independent test set with a sensitivity and specificity of 87% and 85%, respectively. However, the models using barely features extracted from the unsegmented tumor core and the tumor margin predicted the NAC response with lower performance and an accuracy of up to 76.4%. Kaplan–Meier survival analyses showed that the patients predicted as responders using the optimal QUS biomarker demonstrated a statistically significantly better survival compared to those predicted as non-responders.

## Materials and methods

### Study protocol

This study was conducted under the guidelines and regulations in accordance with institutional research ethics board approval from Sunnybrook Health Sciences Centre (SHSC), Toronto Canada. The study was open to all women who were diagnosed with LABC aged 18–85 and planned for NAC followed by surgery. In accord with this, 181 eligible patients were recruited for the study after obtaining written informed consent. A core needle biopsy was performed for all patients to confirm cancer diagnosis, and determine the tumor grade and histological subtype. Also, for each patient pre-treatment magnetic resonance (MR) images of the breast were acquired to determine the initial tumor size. Ultrasound data were acquired from the patients immediately before the start of NAC. Ultrasound scans were performed with patients lying supine with their arms above their heads. Patients were followed up to 10 years after their treatment and their clinical data were recorded for recurrence-free survival analysis. For this study, about 30% of patients (n = 53) were randomly selected and separated to form an unseen independent test set, and the remaining patients (n = 128) were considered as the training set.

### Clinical and pathological response evaluation

All patients had breast surgery after completing their neoadjuvant chemotherapy. For assessing residual tumor size, an MRI scan of the breast was obtained right before the surgery. The surgical specimens were stained with hematoxylin and eosin (H&E) and prepared when possible on whole-mount 5″ × 7″ pathology slides which were digitized using a confocal scanner (TISSUEscope, Huron Technologies, Waterloo, ON). All pathology samples were examined by a board-certified pathologist who remained blinded to the study results. Patients were categorized into two groups of responders and non-responders using a modified response (MR) grading system which was based on response evaluation criteria in solid tumors (RECIST)^49^ and histopathological criteria^37,50^. The MR score was defined as follows: MR 1: no reduction in tumor size; MR 2: up to 30% reduction in tumor size; MR 3: 30% to 90% reduction in tumor size or a very low residual tumor cellularity determined histopathologically; MR 4: more than 90% reduction in tumor; MR 5: no evident tumor and no malignant cells identifiable in sections from the site of the tumor; only vascular fibroelastotic stroma remaining, often containing macrophages; nevertheless, ductal carcinoma in situ may be present. The patients with a MR score of 1–2 (less than 30% reduction in tumor size) and 3–5 (more than 30% reduction in tumor size or with very low residual tumor cellularity) were determined as non-responders and responders, respectively. In accordance with this, 138 and 43 patients were determined as responders and non-responders, respectively.

### Ultrasound data acquisition

Ultrasound data were obtained using an RF-enabled Sonix RP, (Ultrasonix, Vancouver, Canada) system utilizing an L14-5/60 transducer, operating at the center frequency of ~ 6 MHz, and with a -6 dB bandwidth range of 3–8 MHz. For each breast tumor, ultrasound RF data and B-mode images were acquired at four to seven image planes across the breast with approximately 1 cm intervals. An oncologist selected the breast region for ultrasound scanning and determined acquisition scan planes via a physical examination of the patient. The image size along the lateral and axial directions was 6 cm and 4–6 cm, respectively. The focal depth was set at the center of the tumor depending on the individual patient circumstances. The RF data were acquired with a sampling frequency of 40 MHz and digitized with 16-bit resolution.

### Parametric map generation

For generating the QUS parametric images, the tumor core was manually outlined by experts on each scan plane using the associated B-mode image. In addition, the tumor margin contour was automatically generated with a thickness of 5 mm around the core. The parametric maps were generated for all imaging planes of the tumor using a sliding window analysis throughout the entire region of interest (tumor core and margin) with windows of size 2 mm × 2 mm and 95% overlap in both lateral and axial direction.

The QUS spectral analyses were performed to derive MBF, SS, SI, ESD, EAC parameters^26,27^. The power spectrum was calculated using the Fourier transform of the Hanning-gated RF data for every scan line within the analysis window and then averaged. A reference phantom technique was used to normalize the average power spectrum to remove the effects of the system transfer function and transducer beam-forming^51,52^. The reference phantom was composed of 5 to 30 μm diameter glass beads embedded in a homogeneous background of microscopic oil drop- lets in gelatin (Medical Physics Department, University of Wisconsin, USA). The attenuation coefficient and speed of sound parameters of the reference phantom were 0.576 dB/MHz.cm and 1488 m/s, respectively. The attenuation coefficient estimate (ACE) of tumor was calculated using a spectral difference method^51^, and used for attenuation correction of the normalized power spectrum using the point attenuation compensation method. A two-layer (intervening tissue and tumor) attenuation correction was performed using total attenuation estimation^51^. An attenuation coefficient of 1 dB/MHz.cm was assumed for intervening breast tissue based on ultrasound tomography measurements of the breast^53^. The MBF, SS and SI parameters were estimated using a linear regression analysis within the -6 dB bandwidth of the transducer^26,54,55^. The ESD and EAC parameters were derived by fitting a spherical Gaussian form factor model to the estimated backscatter coefficient^56,57^.

### Segmentation of intra-tumor regions

**Figure 1:**
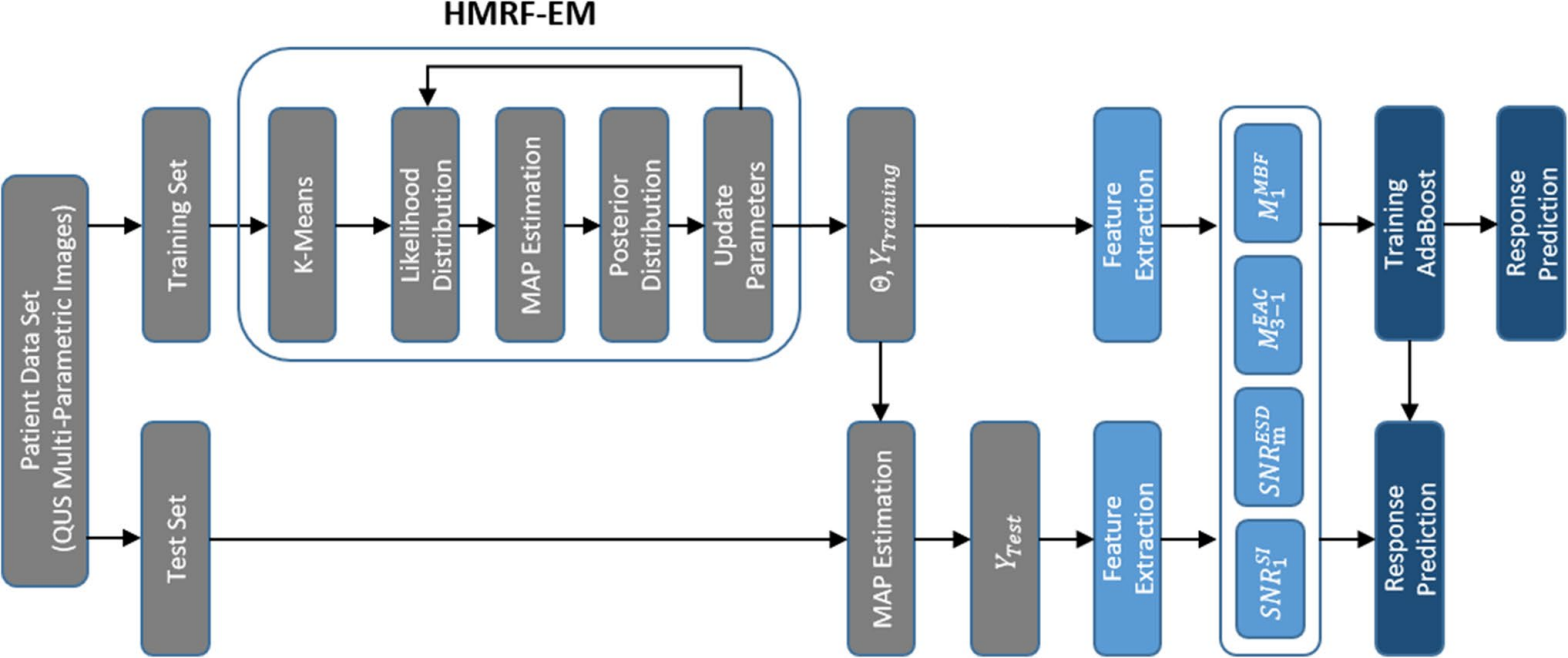
Overall diagram of the proposed framework for identification of intra-tumor regions on QUS multi-parametric images and therapy response prediction.

Figure 1 demonstrates the overall diagram of the proposed framework for segmentation of intra-tumor regions on QUS multi-parametric images that were applied in the therapy response prediction model. The intra-tumor regions were identified at pixel level on QUS parametric images using a HMRF-EM algorithm and the tumor core parametric maps of ESD, EAC, MBF and SI as different data channels (described further below). The optimum number of distinct regions within tumors was determined using the elbow method over the samples of the training set^58^. Specifically, the intra-tumor segmentation was performed for different number of regions, and the Baysian information criterion (BIC) was estimated as the clustering quality metric. Subsequently, the least number of regions associated with a low BIC (the elbow point in the plot of BIC versus different number of regions) was identified. Using this method, the optimum number of distinct intra-tumor regions on the QUS parametric maps was determined as three regions.

A modified HMRF model was trained using an EM algorithm for segmentation of intra-tumor regions^48^. The HMRF-EM is an unsupervised classification method originally proposed for computer vision applications^59^. This method can be adapted for segmentation of multi-channel color images^48^, and medical imaging data^48,60^. In this work, we applied different QUS parametric maps as different channels of data (features) to segment intra-tumor distinct regions. For each pixel *i* (*i* = *1, …, N*), the feature set can be defined as $x_{i} = \left( {I_{i}^{ESD} , I_{i}^{EAC} , I_{i}^{MBF} , I_{i}^{SI} } \right)$. The goal is to infer the labels $Y = \left( {y_{1} , y_{2} , \ldots , y_{N} } \right)$ where $y_{i} = \left\{ {1,2,3} \right\}$, for all tumor core pixels within a set of parametric maps $X = \left( {x_{1} , x_{2} , ... ,x_{N} } \right)$ with maximum a posteriori probability (MAP) estimation. In other words, the estimated labels Y* should satisfy:

1 $$ Y^{*} = argmax_{y} \left\{ {P\left( {X|Y,\theta } \right)P\left( Y \right)} \right\} $$

where the prior probability $P\left( Y \right)$ is a Gibbs distribution and $\theta$ is representative of multivariate Gaussian distribution parameters. For the joint likelihood probability, we have:

2 $$ P\left( {X{|}Y,\theta } \right) = \mathop \prod \limits_{i} P{(}x_{i} {|}Y, \theta ) = \mathop \prod \limits_{i} P{(}x_{i} {|}y_{i} , \theta_{i} ) $$

where $P{(}x_{i} {|}y_{i} , \theta_{i} )$ is a multivariate Gaussian distribution with parameters $\theta_{{x_{i} }} = \left( {\mu_{i} , {\Sigma }_{i} } \right)$, such that $\mu_{i}$ is the mean and ${\Sigma }_{i}$ is the precision matrix of the distribution. The modified HMRF-EM algorithm was used to solve Eq. (1). To estimate initial parameters and labels, a K-means algorithm was applied on the parametric maps. Then, the following steps were performed iteratively:

1. Compute the likelihood distribution $P^{t} (x_{i} |y_{i} , \theta_{i} )$ in step *t*.
2. Use the current parameter set $\theta^{t}$ to estimate the labels by the MAP estimation algorithm^48^.
3. Calculate the posterior distribution $$P^{t} \left( {l{|}x_{i} } \right)$$ for all the $$l$$ ∈ {1,2,3} and all pixels $$ x_{i}$$ utilizing the Bayesian rule.
4. Compute the updated parameter set $\theta^{t + 1}$ with calculated posterior distribution.

In this study, we performed the iteration on the whole training set for 15 times or until convergence. Also, we repeated the inner loop in the MAP estimation algorithm for 10 times or until convergence. Subsequently, the estimated Gaussian distribution parameters (θ) were used to determine the labels of the pixels in the parametric maps of the test set. The segmented regions were numbered based on the mean-value in the MBF parametric maps of the training set from the highest (first region) to the lowest (third region) values. The in-group proportion (IGP) criterion was computed at patient level using the HMRF model developed over the entire training set to evaluate the reproducibility of the clustering results and validate the consistency of the identified intra-tumor regions in both the training and independent test sets^61^.

### Feature extraction and biomarker discovery

A total of 56 features were extracted from the segmented intra-tumor regions and the tumor margin in the QUS parametric maps of ESD, EAC, MBF and SI. The extracted features included mean-value and signal to noise ratio (SNR) of each parametric map within the tumor core (4 × 2 features), mean-value and SNR of each parametric map within the tumor margin (4 × 2 features), mean-value and SNR of each parametric map within each segmented region (4 × 3 × 2 features), the difference between the mean-value of each two segmented regions in each parametric map (4 × 3 features), the proportion area of each segmented region within the tumor core (3 features), and the relative area of the tumor margin to the core. The SNR of each region was acquired by calculating the ratio of the average pixel value to the standard deviation of pixel values of the region, as a measure of spatial heterogeneity^62^. The features were calculated for all 2D imaging planes associated with each tumor and subsequently averaged over the entire tumor volume.

A multi-step feature reduction/selection process was applied to eliminate the redundant and irrelevant features that do not contribute to the predictive model and obtain an optimal QUS feature set for robust response prediction. In the first step, the features were ranked and reduced to 21 features using the minimal-redundancy-maximal-relevance (mRMR) method^55^. In the next step, the final features were selected from the reduced feature set using a sequential forward selection (SFS) method. A five-fold cross-validated accuracy on the training set was used as the criterion in the SFS method with an AdaBoost decision tree model as the classifier^59^. The SFS method selected four features as the optimal QUS feature set (biomarker) that was applied for training the response prediction model.

Four other experiments were conducted for comparison. In the first two experiments, the features extracted from the entire tumor core without considering any intra-tumor regions, and the tumor margin were used for response prediction. Specifically, eight features including the mean-value and SNR of each parametric map within the tumor core (4 × 2 features), and 16 features consisting of the mean-value and SNR of each parametric map within the tumor core (4 × 2 features) and the tumor margin (4 × 2 features) were applied in these experiments, respectively. In both cases, the best feature set was selected using a similar SFS method as described above. The best feature sets included four features in both experiments, and were separately applied for response prediction as described below. In the third experiment, a similar feature reduction/selection procedure was applied on a merged set of standard clinical features and the 56 QUS features derived from the intra-tumor regions and tumor margin to obtain an optimal feature set for response prediction. The clinical features included the initial tumor size, estrogen/progesterone receptor (ER/PR) status, human epidermal growth factor receptor 2 (HER2) status and the age of patient. In the fourth experiment, only the clinical features were used to develop a response predictive model as described below.

### Response prediction and risk assessment

To address the imbalance issue of the dataset, the minority class in the training set was oversampled to the size of the majority class using the synthetic minority oversampling technique (SMOTE)^63^. An AdaBoost decision tree model was adapted for response prediction in each experiment. After training each model on the oversampled training set, its performance was evaluated on the independent test set using the accuracy, sensitivity, specificity, and the area under the receiver operating characteristic (ROC) curve (AUC).

Survival analysis was performed to assess the efficacy of the developed QUS biomarker at pre-treatment in differentiating the LABC patient cohorts with different recurrence-free survival determined many years later. The Kaplan–Meier survival curves were generated for the responders and non-responders identified based on the model’s prediction at pre-treatment, and at post-treatment based on the clinical and histopathological criteria. A log-rank test was used to assess for statistically significant differences between the survival curves of the two patient cohorts (responders versus non-responders).

## Results

The clinical and histopathological characteristics of the participating patients are provided in Table 1. The average age of the patients was 50.6 years. The patients had an average initial tumor size of 5.2 cm, and at the end of their treatment, the average residual tumor size was 2.5 cm. In terms of histology, 90.3% of the tumors were diagnosed with invasive ductal carcinoma, 3.4% with invasive lobular carcinoma, and 6.3% with invasive metaplastic carcinoma. Further, 10.6% of the patients were diagnosed with grade 1 tumors, 38.8% with grade 2 tumors, and 50.6% with grade 3 tumors. At the end of the treatment, 76.2% of the patients were identified as responders, and 23.8% as non-responder, according to the clinical and histopathological criteria.

**Table 1:** Patient characteristics.

Characteristic	Mean ± SD/percentage
**Age**	50.6 ± 11.5 years
**Initial Tumor Size**	5.2 ± 2.7 cm
**Residual Tumor size**	2.5 ± 3.4 cm
**Histology**	
Invasive Ductal Carcinoma	90.3%
Invasive Lobular Carcinoma	3.4%
Invasive Metaplastic Carcinoma	6.3%
**Tumor Grade**	
Grade I	10.6%
Grade II	38.8%
Grade III	50.6%
**Molecular Features**	
ER+	63.4%
PR+	54.7%
HER2+	34.3%
Triple Negative	24.4%
ER+/ PR+/ HER2+	18.6%
ER+/ PR+/ HER2-	33.7%
ER−/ PR−/HER2+	10.5%
**Response**	
Responders	76.2%
Non-Responders	23.8%

**Figure 2:**
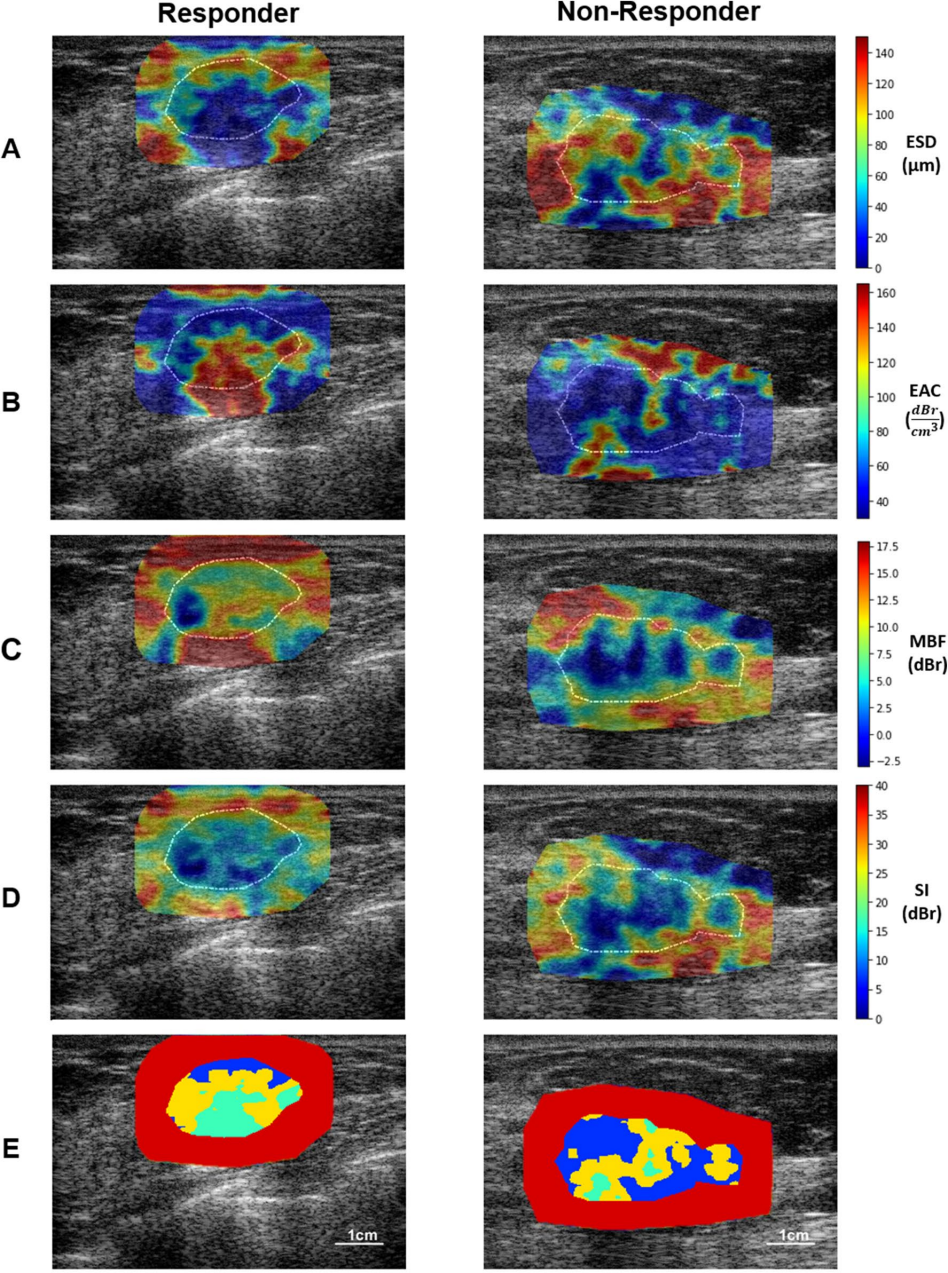
**A**-**D**: Ultrasound B-mode images with parametric overlays of ESD (**A**), EAC (**B**), MBF (**C**), and SI (**D**) acquired from a representative responder and non-responder to NAC. The tumor core has been outlined with white dashed line. (**E**) distinct intra-tumor regions (region 1: green, region 2: yellow, region 3: blue) segmented using the HMRF-EM algorithm, surrounded by the tumor margin area (red).

Figure 2 demonstrates QUS parametric maps of ESD, EAC, MBF and SI overlaid on ultrasound B-mode images acquired from representative responding and non-responding patients, respectively. Distinct intra-tumor regions identified using the HMRF-EM algorithm are presented in Fig. 2E. The QUS parametric maps obtained from the responding and non-responding patients were different in terms of mean and spatial distribution of pixel values. A considerable difference was observed in size, and the mean, distribution and difference of the pixel values of the intra-tumor segmented regions on the QUS parametric maps acquired from the responders and non-responders. An average and median IGP of 0.99 was obtained at patient level in both the training and test sets, implying a high-level of consistency in the intra-tumor regions identified using the HMRF-EM model.

**Figure 3:**
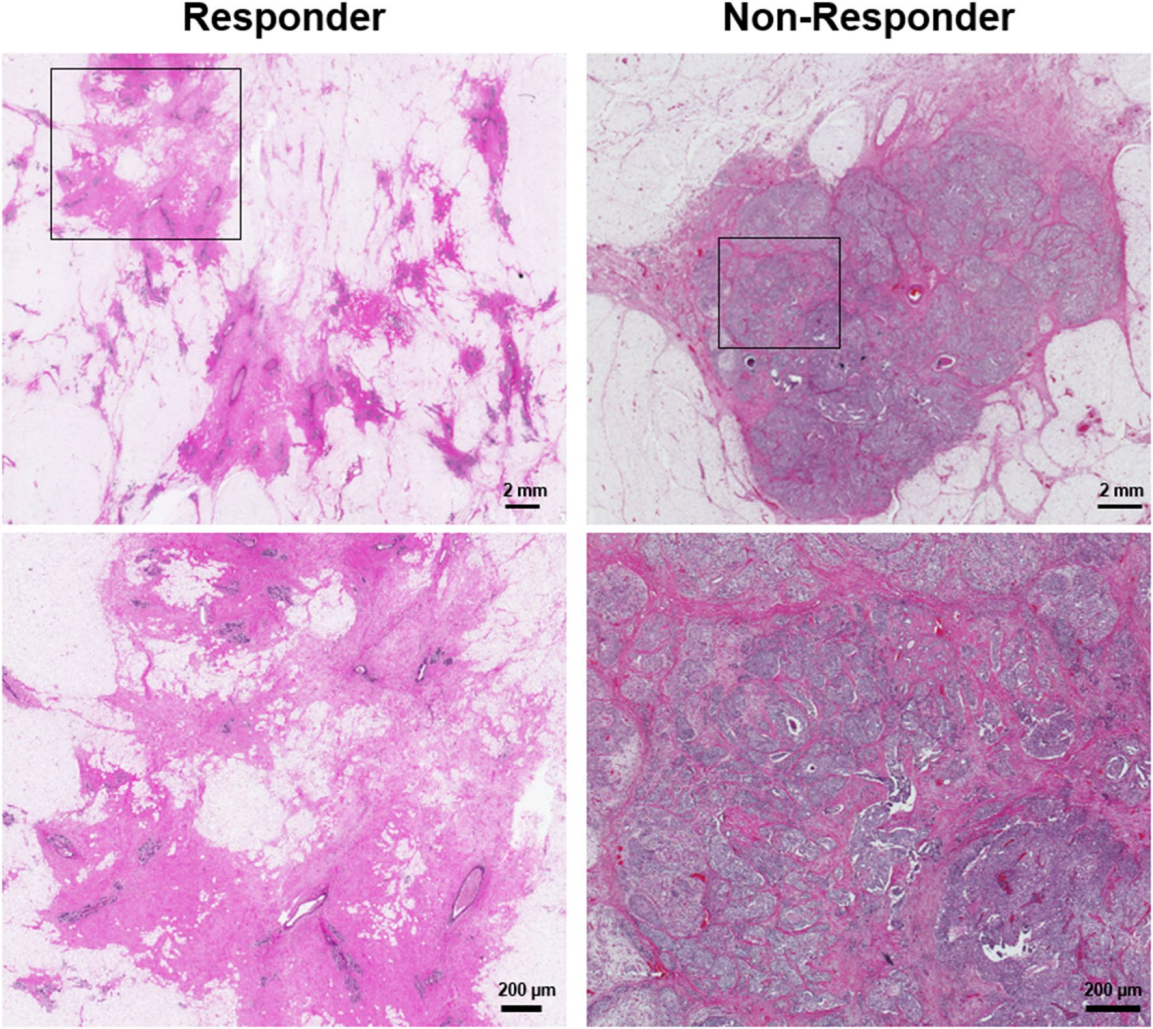
Whole mount histopathology images of mastectomy specimens acquired from representative responding and non-responding patients, at low (top) and high (bottom) magnifications. The scale bars represent 2 mm and 200 µm in low and high-magnification images, respectively.

Figure 3 shows H&E stained histopathology images of the surgical specimens obtained from representative responding and non-responding patients. Whereas the histology images show a large residual tumor in the mastectomy specimen of the non-responding patient, the images acquired from the responding patient demonstrate the tumor bed area with chemotherapy effects and no residual tumor. The images also show considerable heterogeneity within the tumor (bed) area.

**Figure 4:**
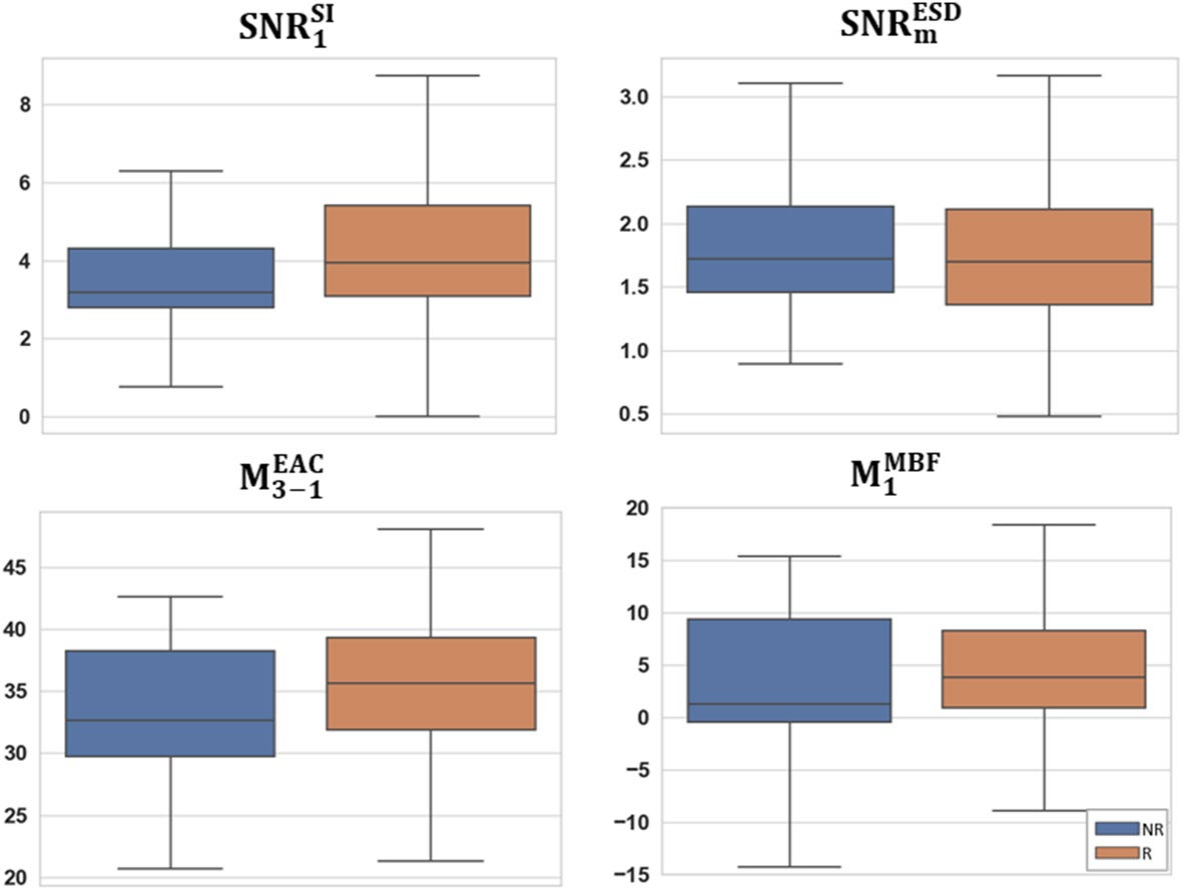
Box plots of the selected features including $$SNR_{1}^{SI}$$, $$SNR_{m}^{ESD}$$, $$ M_{3 - 1}^{EAC}$$, and $$ M_{1}^{MBF}$$ for the responders and non-responders in the training set.

The multi-step feature selection process resulted in a QUS biomarker for NAC response prediction with four features out of 56 features including SNR of the SI parametric map within the first region $$\left( {SNR_{1}^{SI} } \right)$$, SNR of the ESD parametric map within the tumor margin $$\left( {SNR_{m}^{ESD} } \right)$$, difference between the mean-values of the EAC parametric map within the first and third regions $$(M_{3 - 1}^{EAC} )$$, and mean-value of the MBF parametric map within the first segmented region $$ (M_{1}^{MBF} )$$. The first and third regions are associated with the highest and lowest mean-values in the MBF parametric maps, respectively. Figure 4 demonstrates the box plots of the selected features for responders and non-responders in the training set. Whereas various levels of difference can be observed in the selected features between the responders and non-responders, a combination of these features is expected to differentiate better between the response groups at pre-treatment. The impurity-based importance of these features in the trained AdaBoost decision tree model for response prediction was computed as 0.28, 0.26, 0.24, 0.22 for $$SNR_{1}^{SI}$$, $$SNR_{m}^{ESD}$$, $$ M_{3 - 1}^{EAC}$$, and $$ M_{1}^{MBF}$$, respectively.

Applying a similar feature selection method on the eight features derived from the unsegmented tumor core resulted in four features including mean-value of the MBF and SI parametric maps ($$M_{C}^{MBF}$$ and $$M_{C}^{SI}$$), and SNR of the EAC and SI parametric map ($$SNR_{C}^{EAC}$$ and $$SNR_{C}^{SI}$$) within the tumor core. In case of the 16 features derived from the unsegmented tumor core and the tumor margin the best feature set consisted of mean-value of the MBF and SI parametric maps within the tumor core ($$M_{C}^{MBF}$$ and $$M_{C}^{SI}$$), SNR of the SI parametric map within the tumor margin ($$SNR_{m}^{SI}$$), and SNR of the EAC parametric map within the tumor core ($$SNR_{C}^{EAC} )$$. Further, applying a similar feature reduction/selection procedure on the merged set of standard clinical features with the 56 QUS features derived from the intra-tumor regions and tumor margin resulted in the same QUS features selected previously ($$SNR_{1}^{SI}$$, $$SNR_{m}^{ESD}$$, $$ M_{3 - 1}^{EAC}$$, and $$ M_{1}^{MBF}$$) with no clinical feature included in the optimal feature set.

**Figure 5:**
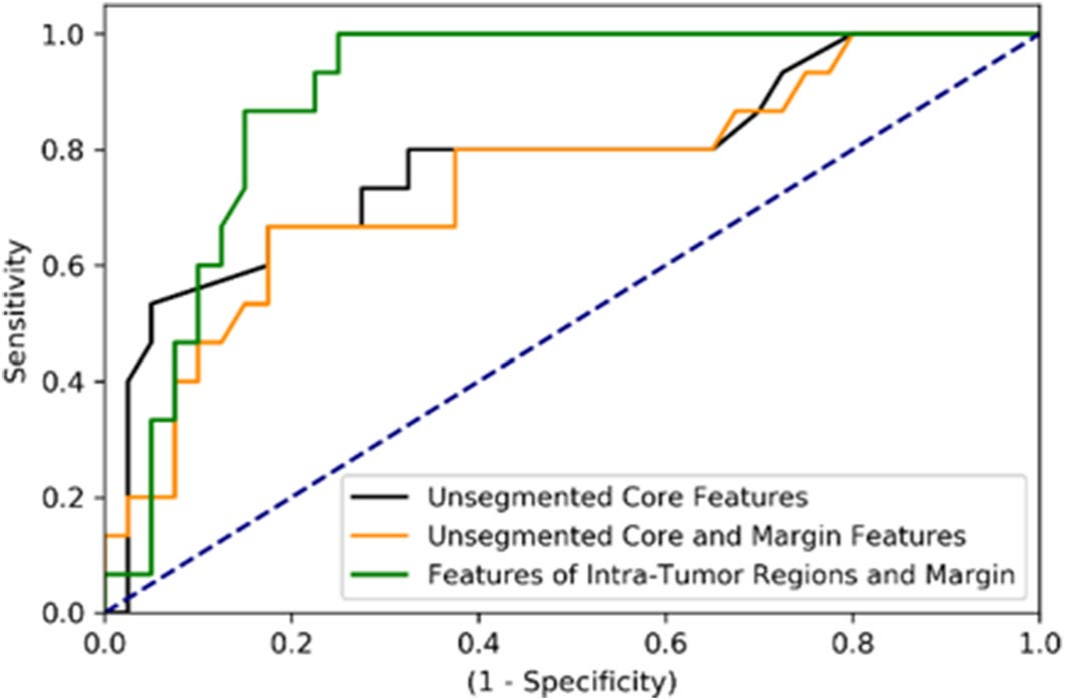
The ROC curves of the therapy response prediction models with different QUS feature sets on the independent test set.

Table 2 presents the result of response prediction on the training and independent test sets using the clinical features and the best feature sets obtained in different experiments. The predictive model with the clinical features could predict the therapy response of patients with a sensitivity, specificity, accuracy and AUC of 40%, 80%, 69.1%, and 0.6, respectively, on the independent test set. Applying the selected QUS features among those extracted from the unsegmented tumor core in response prediction resulted in an accuracy of 74.5%, a sensitivity of 66.6%, and a specificity of 77.5% on the independent test set. Incorporating the QUS features derived from the tumor margin increased the specificity to 80% and the accuracy to 76.4%. Applying the QUS biomarker consisting of the features derived from the segmented intra-tumor regions and the tumor margin resulted in the best performance of the response prediction model on both the training and test sets, with an accuracy, sensitivity, specificity, and AUC of 85.4%, 86.6%, 85.40%, and 0.89, respectively, on the independent test set. Figure 5 demonstrates the ROC curves of the predictive models with different feature sets on the test set.

**Table 2:** Results of response prediction on the training and independent test sets using different clinical and QUS feature sets. Acc: Accuracy; Sen: Sensitivity; Spec: Specificity.

Feature set	Training set				Test set			
	Acc	Sen	Spec	AUC	Acc	Sen	Spec	AUC
Clinical Features: Tumor Size, ER/PR, HER2, Age	77.0%	51.9%	83.8%	0.678	69.1%	40.0%	80.0%	0.6
Unsegmented Core: $M_{C}^{MBF}$, $SNR_{C}^{EAC}$, $SNR_{C}^{SI}$, $M_{C}^{SI}$	81.0%	81.5%	80.8%	0.853	74.5%	66.6%	77.5%	0.79
Unsegmented Core and Margin: $M_{C}^{MBF}$, $SNR_{m}^{SI}$, $SNR_{C}^{EAC}$, $M_{C}^{SI}$	81.0%	77.8%	81.8%	0.86	76.4%	66.6%	80.0%	0.76
Intra-Tumor Regions and Margin: $$SNR_{1}^{SI}$$, $$SNR_{m}^{ESD}$$, $$ M_{3 - 1}^{EAC}$$, $$ M_{1}^{MBF}$$	90.5%	88.9%	91.0%	0.974	85.4%	86.6%	85.0%	0.89

The best value in each column is underlined

**Figure 6:**
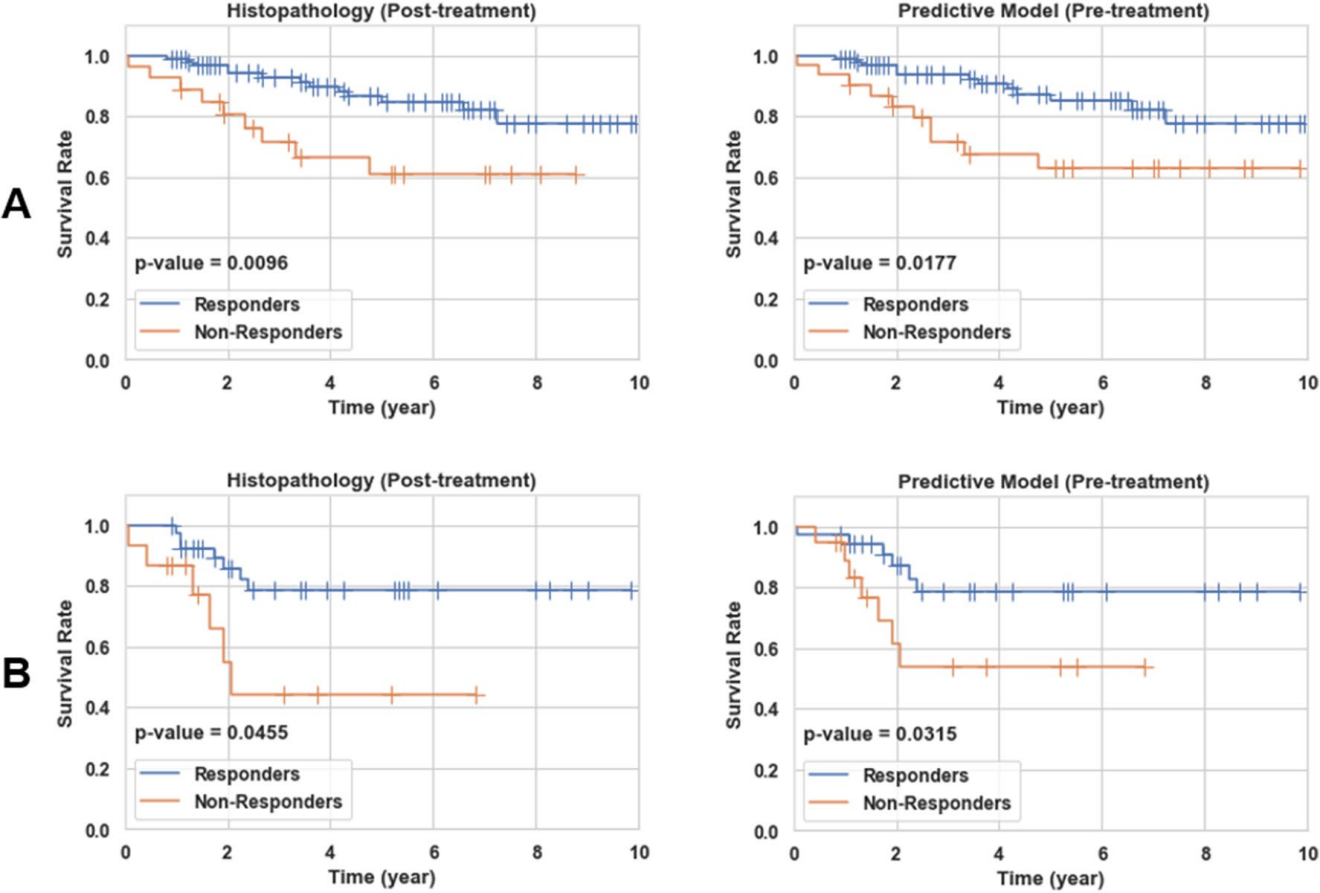
Ten-year recurrence-free survival curves for responding and non-responding patients in the training (**A**) and independent test set (**B**) identified at post treatment based on the clinical and histopathological criteria, and at pre-treatment using the developed predictive model with the optimal QUS biomarker.

Figure 6 demonstrates the ten-year recurrence-free survival curves for the responding and non-responding patients identified based on prediction at pre-treatment using the optimal QUS biomarker, and at post-treatment using the clinical and pathological criteria. Statistically significant differences (p-value < 0.05) were observed between the survival curves of the histopathological response cohorts in both the training and test sets, and responders demonstrated a significantly higher survival rate compared to non-responders. Similar trends were observed in the survival curves of the response cohorts predicted at pre-treatment, with statistically significant differences between the long-term survival of the two cohorts in both the training and test sets.

## Discussion

In this study, a novel method was investigated to predict breast cancer response to NAC using the characteristics of distinct intra-tumor regions on QUS multi-parametric images acquired at pre-treatment. A modified HMRF-EM algorithm was applied to segment the intra-tumor regions on QUS parametric images acquired from LABC patients. Several features were derived from the segmented QUS multi-parametric images to characterize the identified intra-tumor regions and tumor margin. A hybrid QUS biomarker consisting of four features were constructed through a multi-step feature selection process for NAC response prediction. Results indicated that the developed QUS biomarker in conjunction with an AdaBoost decision tree model could predict the response of LABC patient to NAC before starting the treatment with an accuracy of 85.4% and an AUC of 0.89. In comparison, applying the best features extracted from the whole tumor core and the tumor margin on the QUS parametric images resulted in an accuracy of 76.4% and an AUC of 0.76. Obtained results support the idea that using characteristics of distinct intra-tumor regions identified on the QUS multi-parametric images can improve the accuracy of therapy response prediction in breast cancer. Further, the results indicate a very good potential of these QUS features in comparison with standard clinical factors for therapy response prediction at pre-treatment. Recurrence-free survival analyses were performed to evaluate the performance of the developed predictive model in differentiating patients in terms of long-term treatment outcomes. The ten-year recurrence-free survival curves obtained for the responders and non-responders identified based on prediction at pre-treatment were very similar to their counterparts generated for the two response groups identified at post-treatment using the clinical and histopathological criteria. Statistically significant differences were observed between the survival of the responders and non-responders identified based on the both methods.

A number of recent studies has demonstrated the potential of QUS spectral and textural parameters to predict and monitor response of breast cancer to chemotherapy^34–37^. Those studies evaluated the efficacy of QUS parameters for therapy response evaluation using a leave-one-patient-out cross-validation approach due to the relatively small size of their dataset. Whereas cross-validation approaches are commonly used in evaluating classification models when limited data are available, they may overestimate a model’s performance due to overfitting. The study here introduced a new method for analyzing the QUS multi-parametric images for response prediction by quantifying the properties of intra-tumor regions. The developed QUS biomarker was evaluated on an independent test set that was kept unseen during the biomarker discovery and predictive model training. The results obtained in this study extend the findings of previous studies on efficacy of QUS parameters for therapy response evaluation to new robust features that were assessed rigorously on independent data.

The results of this study indicated that characteristics of intra-tumor regions and tumor margin identified on QUS parametric maps of ESD, EAC, MBF and SI could be used for predicting the LABC response to chemotherapy prior to start of treatment. These parametric maps provide complementary information regarding the tumor microstructure by quantifying the properties of underlying acoustic scatterers including their size, density, distribution and impedance mismatch^22,23,26^. Recent studies have demonstrated the potential of the QUS parametric maps in characterizing the tumor aggressiveness and its responsiveness to systemic therapies^34,64^. Segmenting the distinct regions on these multi-parametric maps potentially facilitates effective characterization of intra-tumor heterogeneity. Among the three intra-tumor regions identified in this study, region one is associated with the highest MBF and EAC mean-values, and the lowest ESD and SI mean-values in parametric maps of the entire training set, whereas region three has the highest ESD and SI mean-values, and the lowest MBF and EAC mean-values. An accurate interpretation of these regions in terms of tumor biology requires pre-treatment histology or molecular imaging. However, based on the biophysical characteristics of these regions from the QUS multi-parametric images, region one and three can potentially be linked to the necrotic/apoptotic and active tumor areas, respectively. Region two may be associated with edema and highly perfused regions within the active tumor. Spatial heterogeneity within tumor has demonstrated a crucial role in its responsiveness/resistance to anti-cancer therapies and clinical outcome^39^. The findings of this study are in agreement with observations of previous studies that show quantification of tumor heterogeneity and intra-tumor regions (habitats) on imaging is effective for tumor characterization and therapy outcome prediction^27,40,47,65,66^, and encourage investigation of such methodology when co-registered multi-parametric and/or multi-modal imaging data are available for solid tumors.

The optimal QUS biomarker developed through a multi-step feature selection process consists of four features including $$SNR_{1}^{SI}$$, $$SNR_{m}^{ESD}$$, $$ M_{3 - 1}^{EAC}$$, and $$ M_{1}^{MBF}$$. The SNR features in the biomarker can be linked to spatial heterogeneity in tissue microstructure within the associated region. $$ M_{3 - 1}^{EAC}$$ quantifies the difference in density and impedance mismatch of acoustic scatterer in the first and third segmented regions that can potentially be associated with the necrotic/apoptotic and active tumor areas, respectively. $$ M_{1}^{MBF}$$ is associated with the echogenicity of the first intra-tumor region and is related to the size, density and distribution of acoustic scatterers in tissue micro-structure^67^. The selected features imply that the four QUS parametric maps provide complementary information about the responsiveness of breast tumors to chemotherapy, as all the four parametric images have contributed to the developed biomarker with a relatively similar importance in the final predictive model. Further, the features derived from the distinct intra-tumor regions may better characterize a tumor in terms of therapy response as the feature selection algorithm prioritizes those over the features derived from the entire tumor core. The second feature in the biomarker ($$SNR_{m}^{ESD}$$) is a measure of signal quality (homogeneity) in effective scatterer dimeters within the tumor margin. This is in agreement with the findings of previous studies that reported the importance of tumor margin characteristics in diagnostic and prognostic applications^37,66,68,69^. Whereas the intra-tumor segmentation strategy is potentially expected to improve the feature robustness with respect to variations in the tumor contour, such robustness should be evaluated rigorously in future studies using multi-observer and/or automatically segmented tumor contours. Future studies may also adapt other methods proposed for clustering tumor habitats^43^ to identify intra-tumor regions on QUS multi-parametric images and investigate their performance in comparison with the HMRF-EM algorithm for tumor characterization and therapy outcome prediction.

In conclusion, this study demonstrated that intra-tumor regions in LABC could effectively be segmented on QUS multi-parametric maps for chemotherapy response prediction. The QUS biomarker developed using this methodology could predict the breast tumor response to NAC with high sensitivity and specificity and classify patients into two cohorts with significantly different long-term outcomes. Predicting cancer response to chemotherapy at pre-treatment with demonstrated correlations to long-term survival can facilitate adoption of precision medicine for cancer patients. The results obtained in this study are encouraging. However, studies on larger patient populations are required to assess further the efficacy and robustness of the proposed methodology in clinic. Availability of data from larger patient cohorts will also permit stratified analyses across breast cancer sub-types to explore the performance of the developed models for different molecular and histological sub-types.

## Data Availability

Data were collected and available at the Odette Cancer Centre, Sunnybrook Health Sciences Centre, Toronto, ON, Canada.
